# PVA-PDMS-Stearic acid composite nanofibrous mats with improved mechanical behavior for selective filtering applications

**DOI:** 10.1038/s41598-018-34440-5

**Published:** 2018-10-30

**Authors:** Shama Perween, Ziyauddin Khan, Somendra Singh, Amit Ranjan

**Affiliations:** 1 Rajiv Gandhi Institute of Petroleum Technology, Jais, Uttar Pradesh India; 2 School of Energy and Chemical Engineering, Ulsan National Institute of Science and Technology (UNIST), Ulsan, Republic of Korea; 3 Present Address: Laboratory of Organic Electronics, Department of Science and Technology, Linköping University, SE-60174 Norrköping, Sweden

## Abstract

In this work, we report a facile way to fabricate composite nanofibrous mats of polyvinyl alcohol (PVA), polydimethylsiloxane (PDMS), and stearic acid (SA) by employing the electrospinning-technique, with PDMS fraction ranging from 40w% to nearly 80w%. The results show that for a predetermined fraction of PVA and SA, incorporation of an optimal amount of PDMS is necessary for which the mats exhibit the best mechanical behavior. Beyond this optimal PDMS fraction, the mechanical properties of the composite mats deteriorate. This result has been attributed to the ability of the SA molecules to mediate binding between the PVA and PDMS long-chain molecules *via* van-der-Waals bonding. The morphological, structural, mechanical, and thermal characterizations respectively using SEM, XRD, DMA/tensile test, and DSC lend support to this explanation. By this method, it is possible to control the hydrophilicity/oleophilicity of the mats, and the mats show an excellent selective permeability to oil as compared to water and successfully filter water from a water-in-oil emulsion. Incorporation of SA not only serves to aid in electrospinning of a PDMS-rich nanofibrous mat with good mechanical strength and control over hydrophilicity/oleophilicity, but also has a potential use in fabricating sheets impregnated with phase change materials for thermal energy storage.

## Introduction

Electrospinning is a simple yet versatile tool to produce ultrathin nanofibrous mats and enables use of nanotechnology in various applications^1–5^. A composite nanofibrous mat with desirable properties can be fabricated by judiciously preparing the spinning solutions. However, some highly desirable materials such as PDMS are extremely difficult to be electrospun. Polydimethylsiloxane (PDMS) is a well reported elastomer with high transparency, biocompatibility, chemical inertness, non-flammability, and non-toxicity. It has wide applications in diverse areas including biology^6^, microfluidics^7–11^, energy-storage devices^12,13^, and sensors^14–17^. However, in most applications PDMS is used in the form of either a continuous film or microfluidic channels. Very limited work has been reported on PDMS fibers due to the difficulties in preparation of PDMS fibers using conventional fiber-making techniques^18–24^. PDMS, due to having extremely low *T*_g_ (glass transition temperature) and short-length chains, cannot be electrospun in neat form, and often requires to be composited with another component. For example, Yang *et al*.^23^ utilized mixture of PMMA with PDMS where PMMA aided the electrospinning process. PVA is a semi-crystalline polymer easily soluble in water, and unlike PDMS, is easily spinnable. But applications of PVA based nanofibrous mats are severely limited due to their high solubility in aqueous media. A method able to successfully control and balance the hydrophilicity and oleophilicity of the as-spun mats will be highly desirable for producing selectively permeable membranes, and forms the first motivation behind this work. With this target application in mind, we have used stearic acid to mix the PVA and PDMS which are respectively hydrophilic and oleophilic polymers, in various degrees, to prepare electrospinning solutions and obtain their composite nanofibrous mats.

Stearic acid has been frequently used as a phase-change material (PCM) for thermal energy storage applications as it has high melting enthalpy at relatively accessible temperature^25–28^. Stearic acid in a mixture of PVA and PDMS is not only expected to improve the mixing and homogeneity of the spinning solution, and thereby the quality and mechanical strength of the resulting mats, but can also impart multi-functionality to the produced composite mats by acting as a component for thermal energy storage. In a prior work^29^, fabrication of a nanofibrous sheets using electrospinning technique incorporating *mixture*s of fatty acids (stearic and lauric acids) and polyvinyl alcohol was reported. PVA acted as a guiding polymer for electrospinning forming the fibrous matrix and fatty acid served as a phase change material. It was demonstrated that these sheets can act like flexible thermoregulating enclosures. A significant drop in eutectic temperature in the fatty acid binary mixtures was observed when incorporated in PVA nanofibrous mats as compared to their bulk mixtures. However, the nanofibrous sheets incorporating the eutectic composition showed poor mechanical strength and severely limited applications of these mats in thermal energy storage applications. This forms another motivation for the present work.

In our previous work^29^, application of regular solution model to a mixture of lauric and stearic acids inside PVA mats led us to hypothesize that the drop in eutectic temperature in the fatty acids binary mixtures may be attributed to a constrained environment of hydrophilic nature (due to PVA) which alters the molecular interactions between the two components. Incorporating PDMS will also serve to test this conjecture as it will reduce (enhance) the hydrophilicity (oleophilicity) in the nano-fibrous environment. A more detailed study to this end will be presented in a separate work. In this work, we present the fabrication and characterization of these composite nanofibrous mats, and demonstrate their potential application as a selective filter to separate a water-in-oil emulsion. Electrospun nanofibrous films of PVA-PDMS with pure stearic acid with varying compositions have been studied. We have varied only the PDMS content in this work so as to increasingly impart oleophilicity to the membranes. In view of the fact that electrospinning of the pure PDMS is difficult to achieve owing to its low *T*_g_, increasing the PDMS fraction also serves the purpose of fabricating electrospun mats rich in PDMS. PDMS imparts improved mechanical strength to these sheets and renders them hydrophilic/hydrophobic to various degrees in a controllable manner. Thermal, mechanical, and morphological characterizations of these composite nanofibrous sheets of stearic acid-PVA-PDMS have been performed using DMA, tensile tests, differential scanning calorimetry, surface profilometry, and SEM. It was observed that PDMS component improves mechanical strength but only when incorporated up to an optimal concentration. The optimal sample showed excellent selective permeability to oil as compared to water, owing to the chemical nature of PDMS and the nano-fibrous morphology of the membrane mats. Incorporating PVA in the mats enables control over the relative permeability of the membrane towards oil and water.

## Experimental

### Materials

Polyvinyl alcohol (PVA) of M_w_ ranging from 85000 to 1,24,000, used as the guiding polymer for electrospinning, and stearic acid (SA) with 95% purity, were purchased from SD Fine Chem. Ltd. (SDFCL, Bombay, India) and were used without further purification. We have used Sylgard-184 from Dow Corning, Midland, MI, USA as our working cross-linked polydimethoxysilane (PDMS) elastomeric material. Tetrahydrofuran (THF, 99.5%) was purchased from SDFCL and used as a solvent to swell the cross-linked PDMS without cross linkers. Absolute methanol (99.0%) was purchased from SDFCL. DI-Water was used to make all PVA solutions and emulsions.

### Preparation of spinning solutions

To ensure the fabrication of electrospun nano-fibrous composite mat by using electrospinning technique, a solution must be homogenous, stable and have good spinnability. Here, aqueous PVA was chosen as the guiding polymer solution because of its excellent electro-spinnability. The use of water-soluble polymer avoids using toxic organic solvents and the fatty acid constitutes a renewable, nontoxic component with high heat enthalpy^30^. THF was used as a solvent to dissolve PDMS precursor without cross-linker. Three separate spinning solutions of PVA in DI-H_2_O, stearic acid (SA) in methanol and PDMS in THF were prepared. Firstly, 10% PVA solution was prepared by mixing and stirring it in DI water for 3 h at 60 °C and then left to cool until the solution reached the room temperature (RT). Then the second solution of stearic acid (SA) in methanol (4% by weight) was prepared at room temperature separately. The third solution of PDMS without cross-linker was prepared in THF solvent with variable weight percent (w%) of 10%, 15%, 20%, 30%, 40% and 50%) at RT. Thereafter, 10 mL SA solution was added to the PVA solution stirring simultaneously for 2 h so as to obtain a homogenous solution of PVA-SA. This was followed by addition of 10 mL PDMS solution over the next couple of hours. PDMS solutions of variable % weight were added into the PVA-SA solution to prepare six final spinning solutions with varying amount of PDMS. The resulting PVA-SA-PDMS electrospun nanocomposite mats are referred in this paper in an abbreviated form as ENCF. A sample nomenclature table with corresponding sample details is presented in Table 1.

**Table 1:** Sample nomenclature and their compositions.

Samples Weight	ENCF-1	ENCF-1.5	ENCF-2	ENCF-3	ENCF-4	ENCF-5
PVA:SA:PDMS	1:0.4:1	1:0.4:1.5	1:0.4:2	1:0.4:3	1:0.4:4	1:0.4:5
%Weight of PDMS in the as-spun mat	41.66	51.72	58.82	68.18	74.07	78.12

### Preparation of water-in-oil emulsion for membrane filtration application

We have prepared oil rich emulsion (water-in-oil) with oil-water ratio of 7:3 by adding 50 mg of cetyltrymethyl ammonium bromide (CTAB) as a surfactant to 10 mL of the water-diesel oil mixture and leaving the mixture under magnetic stirring for 2 h at room temperature. This yielded a homogenous dispersion of emulsion.

### Characterizations

#### FE-SEM/EDS/X-ray Diffraction/FTIR

The morphologies were imaged by using Field Emission Scanning Electron (FE-SEM) Microscopy (Zeiss Supra 40). The chemical composition was verified by elemental analysis on a Zeiss electron microscope (accelerating voltage ranged from 5 to 20 kV) equipped with an EDS analyzer. To prevent samples charging, a thin gold coating was sputtered onto the samples prior to the analysis. X-ray diffraction data were assessed by using a PANalytical X’pert Pro MPD diffractometer with monochromatic Cu Kα radiation (λ = 1.54056 Å) to investigate the crystallinity. The electrospun as deposited nano-fibrous composite mats were directly used to analyze the sample. The Perkin Elmer Spectrum Two FT-IR spectrometer was used to record the FT-IR spectra of the electrospun as deposited nanofibrous composite mats. All the samples were recorded by using “attenuated total reflectance” (ATR) mode. The PIKE MIRacle single reflection horizontal ATR accessory equipped with a ZnSe ATR crystal was used for recording the FT-IR spectra.

### Mechanical characterization

The tensile test studies were performed by using an H25KS UTM Tinius Olsen extensometer. The tensile strengths of the rectangular (length: 23 mm, width: 6 mm, thickness: 0.04 mm to 0.08 mm) strip samples were recorded at room temperature using a 1000 N load cell and at a crosshead speed of 1.0 mm/min so as to maintain the quasi-static loading conditions. This is important for soft materials as high strain rates can cause viscoelastic effects^31^. The samples are gripped at both ends with “lath-like” clamp with one end fixed and the other end moving. The Young’s moduli of the samples were determined from the linear region (Hookean slope) of the stress versus strain plot and compared amongst all PDMS compositions. The ultimate tensile strength (UTS) is taken at the highest point of the stress–strain curve. The strain at break is taken as the percent elongation just before the fracture of the film. Toughness of the sheets which is a measure of energy that the sheets can sustain prior to failure were determined by integrating the stress-strain curves.

### Dynamic Mechanical Analysis

The dynamic mechanical analysis (DMA) was performed on a DMA Q-800 (Manufacturer: TA Instruments) using the tension mode for the temperature sweep experiments. ASTM D4065-01 norm was followed to record the samples. The specimen samples of 5 mm width and 15 mm length were used for this purpose. The storage (G′) and loss modulus (G″) data under variable temperature from 25 °C to 110 °C with a heating rate of 3 °C/min were recorded at constant frequency (1 Hz).

### Differential Scanning Calorimetry

The differential scanning calorimetry (DSC) data of the samples were recorded on a DSC Q200 from TA Instruments under N_2_ atmosphere (50 mL/min). Pre-weighed (~1.5 mg) as-deposited electrospun fibrous samples were taken in a Tzero Aluminum pan for the measurement. The samples were heated from 24 to 100 °C, cooled to 24 °C, and again heated to 100 °C at a rate of 2 °C/min. The data obtained from the first and the second heating traces of the samples are reported in this article.

### Contact angle measurements

The evaluation of the surface wettability of ENCF fibers towards water and silicone oil were performed via static contact angle measurements using a Drop Shape Analyzer DSA100 Krüss Advance. Three different samples were used for each condition and the mean value is reported here. Deionized water and silicone oil (10 μL) were dropped on the PVA-PDMS-SA ENCF, and the affinity of the drop for the surface towards both water and oil were measured using the circle fitting method. All samples were maintained in a standard room environment (20 °C and 30–35% humidity).

### Electrospinning (Emulsion Electrospinning Technique)

Nanofibers mats were fabricated by using the mixture solution of PVA, SA and PDMS with variable PDMS by weight percent in electrospinning setup purchased from E-Spin Nanotech Pvt. Ltd., India. The spinning solution was filled in a 10 mL plastic syringe having a stainless steel needle at the tip. During the electrospinning, a positive high voltage of 12 kV was applied to the needle, and the fibers were collected on an aluminum foil wrapped around an electrically grounded rotating collector drum as cathode. The needle-to-collector distance was kept in range of 10–12 cm and the solution flow-rate was maintained by using a syringe pump. The temperature and the relative humidity of the spinning chamber were respectively (26 ± 0.1)°C and (40 ± 1)%.

## Results and Discussion

### Morphology

**Figure 1:**
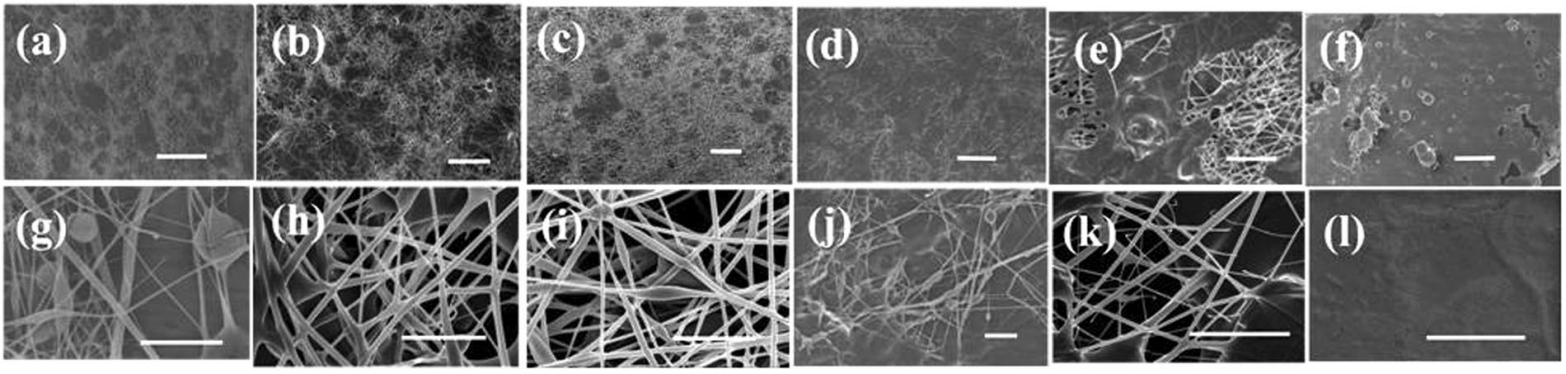
FE-SEM images of ENCF mats (samples ENCF1, ENCF1.5, ENCF2, ENCF3, ENCF4 and ENCF5, from left to right) with increasing concentration of PDMS (41–78%) at lower magnification (**a**–**f**) and at higher magnification (**g**–**l**). Top panel images (**a**–**f**) have scale bar = 50 microns and bottom panel images (**g**–**l**) have scale bar = 5 microns.

**Figure 2:**
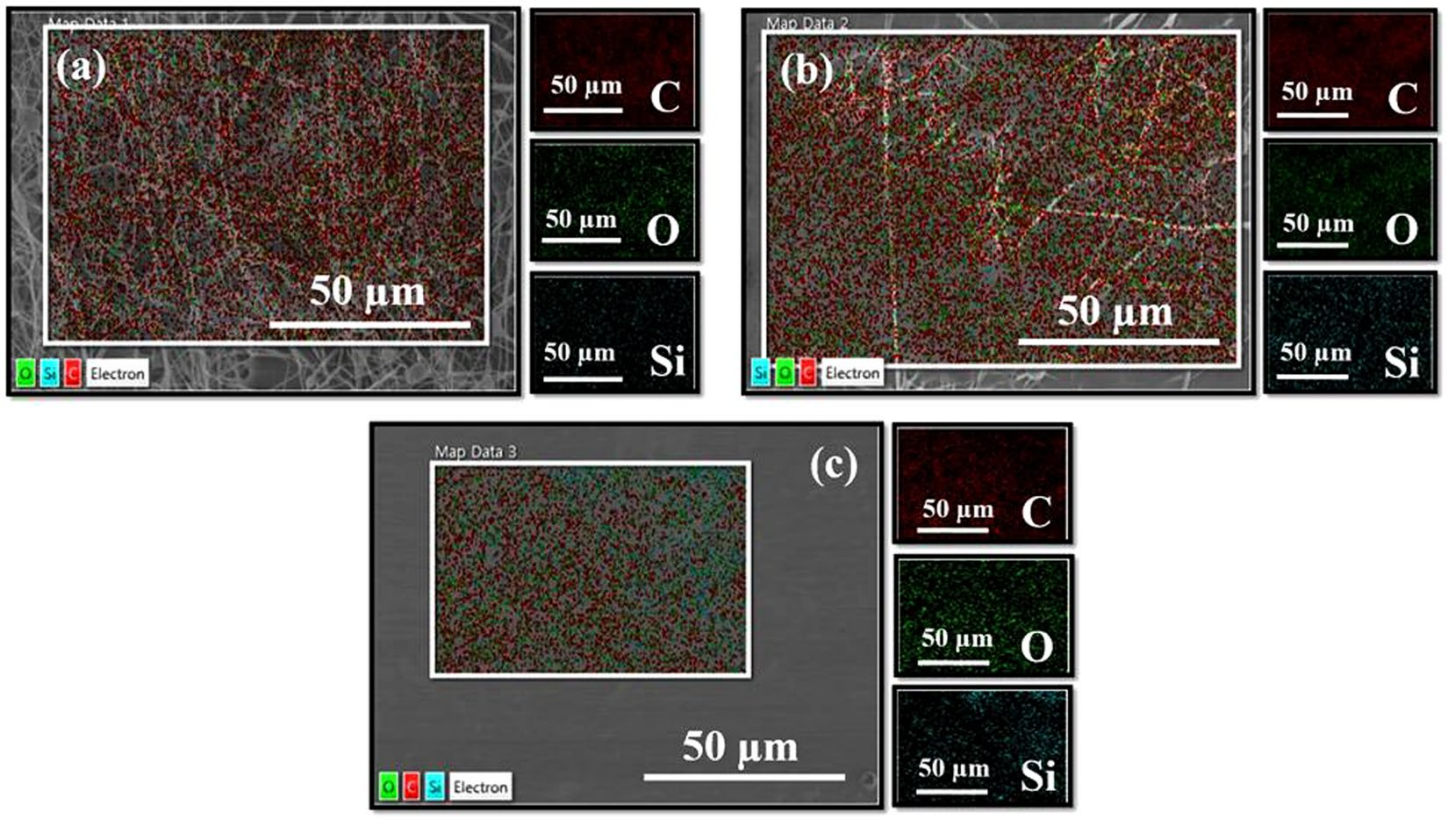
Elemental analysis and mapping of (**a**) ENCF-1 (**b**) ENCF-3 and (**c**) ENCF-5 mats by EDX. Amongst all panels, left sided figure shows the FE-SEM image of the area of interest. Other adjacent sub-panels show elemental maps of other concerned elements, where green dots represent oxygen, cyan green dots represent Si, and red dots represent carbon.

In this section we present the morphological variation in the nanofibrous mats as the fraction of PDMS (without any cross-linker) is increased Fig. 1(a–f,g–l). As observed in the Fig. 1(a–f) at lower magnification; and g-l at higher magnification), ternary sol phase material got successfully electrospun resulting in the formation of smooth and continuous nanocomposite nanofibrous mats (please also see the supplementary material). However, beyond a PDMS weight fraction of 68% the quality of the mats became poorer as the spinnabilty of the solution worsened and the process became partially electrospraying and partially electrospinning. This resulted into mats with fibers immersed in a PDMS rich matrix for higher PDMS fractions as evidenced by the SEM images presented in Fig. 1(f,l). It is to be noted that in these mats the PDMS matrix was un-crosslinked that led to decrease in mechanical strength in mats with higher PDMS content as discussed in a later section. This result also supports the notion that the fatty acids compatibilize the PDMS and PVA but only up to a limiting concentration of PDMS. With the amount of fatty acid fixed in all these samples, beyond a threshold concentration of PDMS, the PDMS molecules are not blended with the PVA, and consequently, are not electrospun. Elemental mapping using EDX presented in Fig. 2 also shows the PDMS phase separation as suggested by the silicon signal spatially segregated from the carbon signal in mats with higher PDMS fraction (ENCF-5).

For samples with PDMS content larger than 78.12% (ENCF-5) solution is still spinnable but the obtained mats were stickier as compared to samples with lower PDMS fractions. The fiber diameters lie in the range of 100–300 nm. Phase separation in spinning solution could be avoided up to a PDMS concentration of 74% (ENCF-4) only beyond which the solution was not stable at longer times (longer than that required for the electrospinning process) and spraying also became effective. The fibers obtained for PDMS fractions less than around 78% (ENCF-5) the mats were completely non-sticky, but samples resulting from spinning solutions with higher PDMS fractions were sticky due to electrospraying leading to presence of a continuous PDMS matrix with fibers immersed within. As argued later and confirmed by various data discussed in this paper, this may be due to the fact the stearic acid molecules, which aid in blending of PVA and PDMS, are in limited supply. As a result, excess PDMS is not incorporated in the fibers and forms electrosprayed matrix in the mats responsible for adhesion exhibited by these mats.

### X-ray diffraction

The X-Ray diffraction studies shed light on the changes occurring in the composite mats as the PDMS concentration is increased. The XRD results help us to propose a model of the molecular processes and arrangement thereof responsible for the observed data. Our FT-IR results done on the as-spun samples confirm no formation of new covalent chemical bonds (please see supplementary data, Fig. S2.). Therefore, the properties of the as spun mats are presumed to be governed by physical interactions among the component molecules.

**Figure 3:**
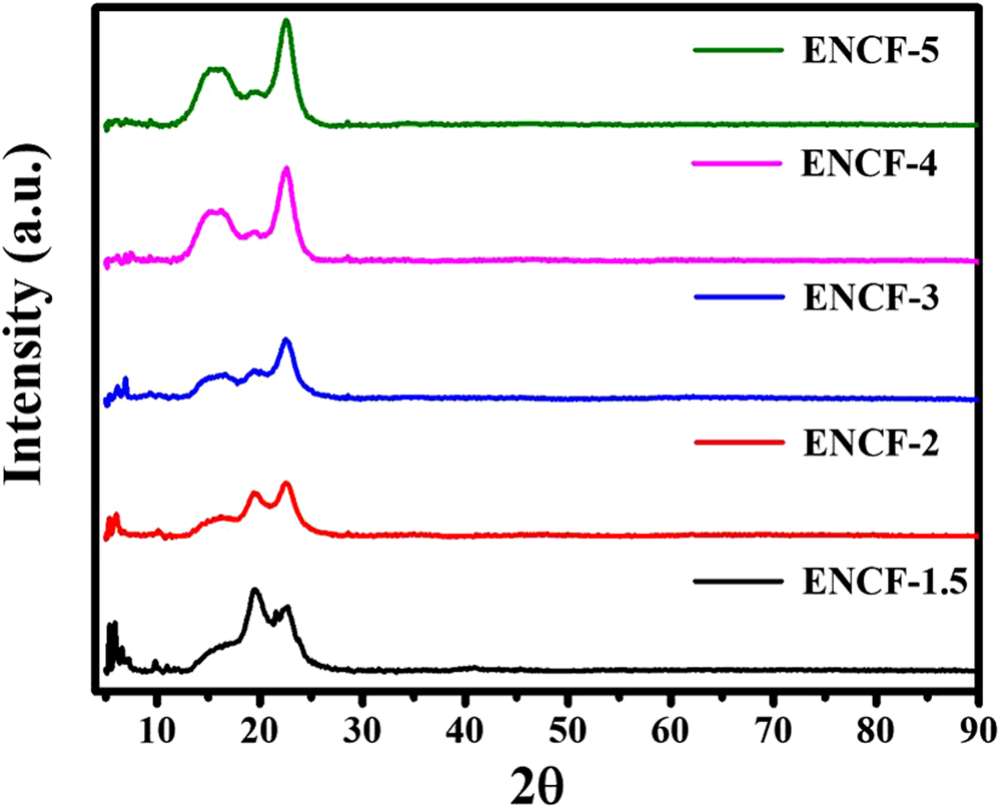
X-ray diffraction patterns of ENCF mats with increasing concentration of PDMS.

The X-ray diffractograms of the mats are presented in the Fig. 3 with increasing concentration of PDMS. Mainly four kinds of peaks are identified which are used for further analysis: (i) the sharp Bragg peaks at 2*θ* values less than 10°, (ii) a broad peak at around 16°, (iii) a peak around 19° and (iv) a relatively sharp peak around 22°. Stearic acid is known to exist in various polymorphs which are named as A, B, C, and E depending on the symmetry of their crystalline arrangements^28,32–35^. The sharp Bragg peaks at 2*θ* values less than 10° are attributed to B and E forms of crystalline stearic acid polymorphs^32^.

The structure and the corresponding unit cell in crystalline PVA have long been recognized^36–39^. Crystalline PVA has pseudo-orthorhombic unit cell showing the most important reflections at 2*θ* of 16°, 19.4°, 20°, and 22.7° with CuK_α_ radiation, which respectively correspond to (001), (101), (10 −1), and (200) planes. The cell parameters are *a* = 7.81 Å, *b* = 2.5 Å, *c* = 5.5 Å. The proposed crystalline structure consists of straight chain pairs of PVA molecules held together by hydrogen bonding and forming a two-layer sheet. These hydrogens bonded two-layer sheets are stacked in parallel and held in place by van-der-Waals attraction. The orientation of the crystallographic axis *a* is along the direction of stacking of double layer sheets, *b* is parallel to the PVA chain, and *c* is mutually orthogonal to other two directions and is parallel to the double-layer sheet.

Our mats show all the major crystalline peaks of PVA, except that the (101) and (10 −1) reflections near 20° are indistinguishable. This suggests an orthorhombic unit cell which has been already reported in crystalline PVA^37^ and is argued to be more favorable in uniaxially drawn PVA fibers. Moreover, the peak at 16° is rather broad and an amorphous hump is also apparent overlapping with this peak. We believe that this broad hump overlapping with the PVA (001) reflection corresponds to amorphous liquid like state of PDMS chains. In experimental and simulation XRD studies on PDMS melt structures, a broad peak has been observed at *q* = 0.85 Å^−1^ (where *q* = 4π sin*θ*/λ) which for CuK_α_ radiation corresponds to 2*θ* ~ 12°. This peak has been attributed to intermolecular correlations in liquid state PDMS chains in bulk^40,41^, where chains are relatively ordered in liquid state. In our case, a nano-scale confinement due to hydrophilic PVA, the intermolecular distance could further be reduced and lead to a reflection at an increased value of 2*θ*. Therefore, we assign a broad peak at around 16° to the combined effect of liquid-like ordering in the PDMS chains and the (001) reflection from PVA.

The following trend in the X-ray diffraction patterns are observed as the PDMS content is increased in our mats: (i) The sharp Bragg peaks at 2*θ* less than 10° due to crystalline stearic acid progressively decrease in strength. (ii) The crystalline peak of PVA at 22° becomes more pronounced. (iii) The crystalline peak near 20° due to (101) reflection becomes less pronounced. (iv) The broad peak at around 16° occurring due to overlap of (001) reflection from PVA and amorphous nature of liquid like ordering in PDMS chains increases in strength. In a study involving adhesion behavior of mixtures of stearic acid and PDMS^42^, the authors found no “weak boundary layer” formation due to surface segregation of stearic acid phase separation, and PDMS is able to dissolve/interact and bind with the stearic acid molecules^43^.

**Figure 4:**
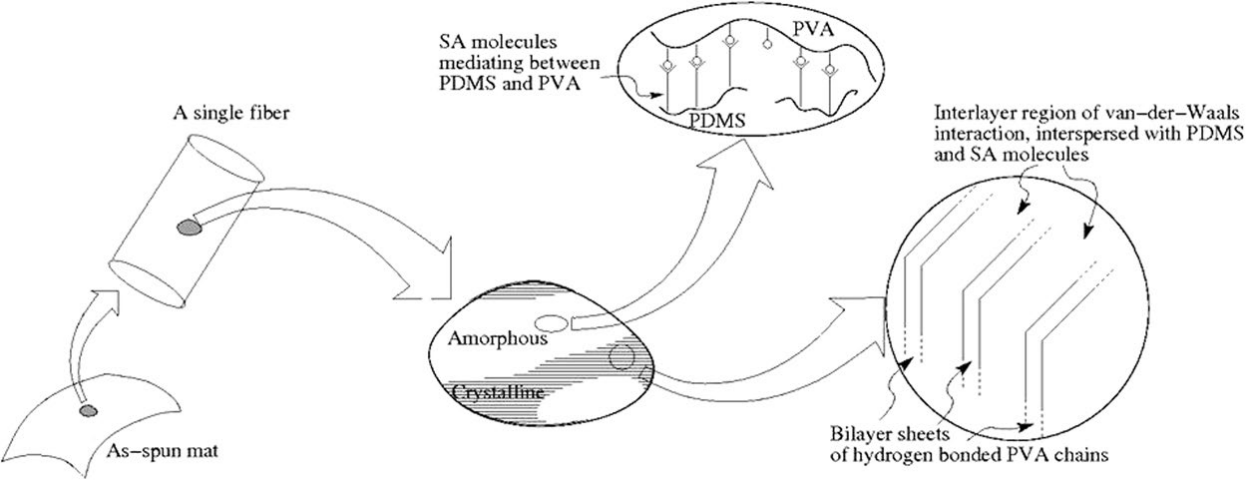
A cartoon of the proposed molecular arrangement amongst the three components of PVA, SA and PDMS molecules.

Based on these observations and prior research findings cited above, we propose the following model for the molecular processes, a cartoon of which is presented in Fig. 4. At smaller PDMS concentration, the mats consist of crystalline PVA with inclusions of crystalline stearic acids. These inclusions lead to reduced mechanical strength, as confirmed by our data on mechanical properties of these mats presented later. The PVA crystals are in the form of stacked double layer sheets. As the PDMS content is increased, the PDMS dissolves stearic acid and disturbs its crystallinity. The PDMS and stearic acid chains penetrate into the van-der-Waals regions between the double layers of hydrogen bonded sheets of the crystalline PVA and also disrupt the hydrogen bonding between the sheets. Since hydrogen bonding is along [101] direction, the (101) reflections become weaker as more PDMS is incorporated in the mats. It can also be argued that the presence of stearic-acid dissolved PDMS in the inter-sheet region reduces the thermal fluctuations in the double layer sheets and thereby enhances the strength of (200) reflection. This model is also in agreement with the studies on mechanical behavior of the mats presented later in the paper. It is to be emphasized here that this model is based on physical considerations only and other tools such as meso-mechanics or molecular dynamic simulations need to be performed to validate the model quantitatively.

These results are in agreement to our earlier proposed physical picture of the molecular process where stearic acid present in crystalline form furnishes SA molecules that bind PDMS to PVA chains via alcohol side groups of the PVA chains through hydrogen and van-der-Walls bonding. As the PDMS fraction reaches a threshold such that all the SA molecules are utilized as the binding links between PDMS and PVA, the crystalline Bragg peaks due to stearic acid disappears. However, due to progressively increasing amount of PDMS experiencing nano-confinement, the fraction of liquid-like ordering in PDMS increases resulting into higher intensity of the broad peak at around 16°. In bulk this broad peak is observed at around 12°, but in our mats, the angle may have increased due to nano-scale confinement leading to more closely spaced chains of PDMS.

### Differential Scanning Calorimetry (DSC)

**Figure 5:**
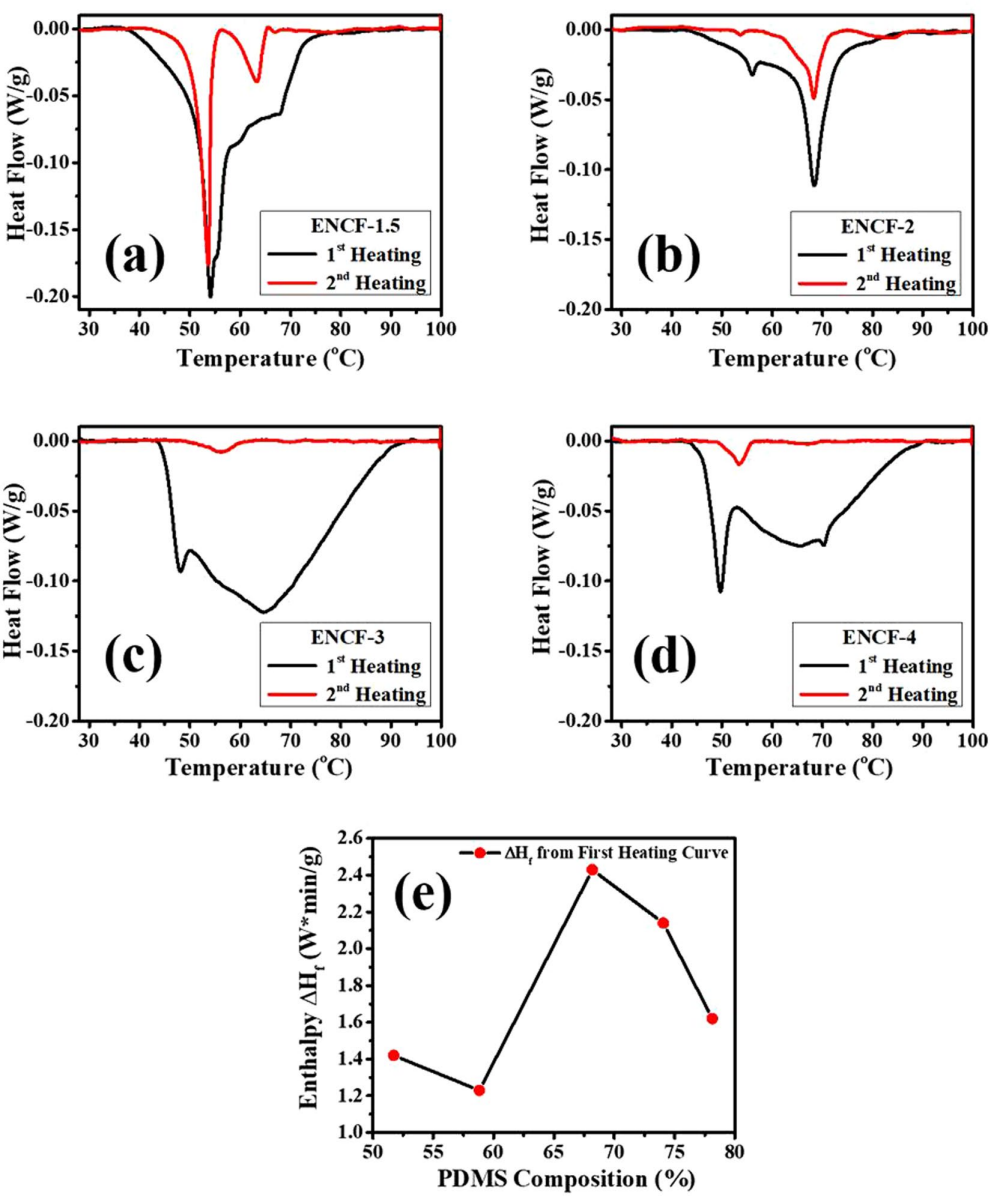
DSC thermograms from first and second heating for (**a**) ENCF-1.5, (**b**) ENCF-2, (**c**) ENCF-3, (**d**) ENCF-4. (**e**) Shows the change in enthalpy over the entire curve during the first heating by measuring the area under the power vs time curve.

DSC traces of the first and second heating scans of samples ENCF-1.5, ENCF-2, ENCF-3, and ENCF-4 are presented in Fig. 5. The following important observations are made in these DSC thermograms. (i) The first heating scan in each sample gives a wide peak (Fig. 5(a)). (ii) The wide peak keeps becoming wider as PDMS fraction is increased up to the sample ENCF-3 beyond which the width of the peak decreases (Fig. 5(b)). (iii) A peak at around 68 °C that disappears in ENCF-3 and ENCF-4. (iv) A peak present around 53 °C which becomes increasingly less pronounced as the PDMS fraction is increased.

The wide peaks observed in the first heating run suggest that the stearic acid crystallites are present with a broad size distribution in the as-spun mats. Width of these peaks increases with increasing PDMS content. The wide distribution could result due to following reason: Larger incorporation of the SA is attained in the PDMS as PDMS fraction is increased. This can cause formation of larger number of composite micro-droplets in the spinning solutions as PDMS content is increased. These droplets would further subdivide under the action of high electrostatic field and subsequently in the mats a larger variation in the micro-crystallites of SA can appear.

The possible transitions in the materials are the melting of stearic acid, solid-solid transformation in stearic acid, and glass transition in PVA. The peak close to 65 °C is attributed to melting of the stable form of the stearic acid. Moreno *et al*.^32^ have performed a detailed study of polymorphs and possible transitions in stearic acid and reported a solid-solid transition which may occur around 55 °C. In all the mats the second heating curve shows a peak at around 53 °C. Singleton *et al*.^33^, have observed solid-solid transition from B to C at 53 °C. The formation of B or C forms depended upon the choice of solvent (benzene or glacial acetic acid) and temperature of crystallization. C form is stable at higher temperatures^28^. Transition from A to C form occurs at 54 °C and B to C at 46 °C^28,34^. Garti *et al*.^35^ obtained B to C transition at 54 °C. Thermodynamic prediction is around 32 °C^44^. The observed difference in the transition temperature has been attributed to a kinetic barrier for the transition to occur^45^. We expect that upon first cooling the stable phase B forms which upon heating transforms to first the C form which then converts to liquid phase. This order of transition however is absent in mats with higher PDMS content (ENCF-3 and ENCF-4). In the mats with lower PDMS content, the stearic acid is present in excess and after some of it is utilized in compatibilizing the PDMS and PVA as discussed earlier in the proposed model of molecular arrangement (Fig. 4), the rest of the stearic acid may crystallize which can show these two transitions. In mats with higher PDMS fraction, only one peak ((having much smaller intensity) is observed. We do not have a satisfactory explanation to this but we conjecture that this may correspond to small amount of left-over stearic acid in metastable A form directly changing to liquid phase. The pathway that converts the metastable A form to liquid and bypasses the formation of stable C form before forming liquid may be taken possibly because of a comparatively lower activation barrier in this pathway. Lowering of the activation barrier at an appropriate temperature in turn may arise due to presence of other species. This conjecture also conforms to our suggested model since a clear solid-solid and solid-liquid transitions are absent in the samples with higher PDMS content. Above the glass transition temperature of PVA, the PVA chains are more mobile and the stearic acid micro-crystallites have also melted. Therefore, the stearic acid is expected to distribute more uniformly after first heating scan and result into less broadened peaks in the second heating scan as seen in our data (Fig. 5).

Figure 5(e) presents the total enthalpy in the first heating given by the area under power vs time curves. We note that the sample ENCF-3 shows highest enthalpy change during the first heating. We argue that this may be possible because dispersion of stearic acid micro-crystallites also functions as a binding agent between the PVA and PDMS molecules. This increases their cohesion and thereby increases their enthalpies per unit mass. Broad DSC thermograms shown by these mats suggests that they can be exploited for one-time thermally triggered delivery of such molecules which can be encapsulated by stearic acid such as drugs which are insoluble in water^46^.

### Mechanical properties

Mechanical characterizations of the mats have been performed by tensile testing experiments and dynamical mechanical analysis. In the subsequent discussion we present results from both of these separately.

### Tensile test

**Figure 6:**
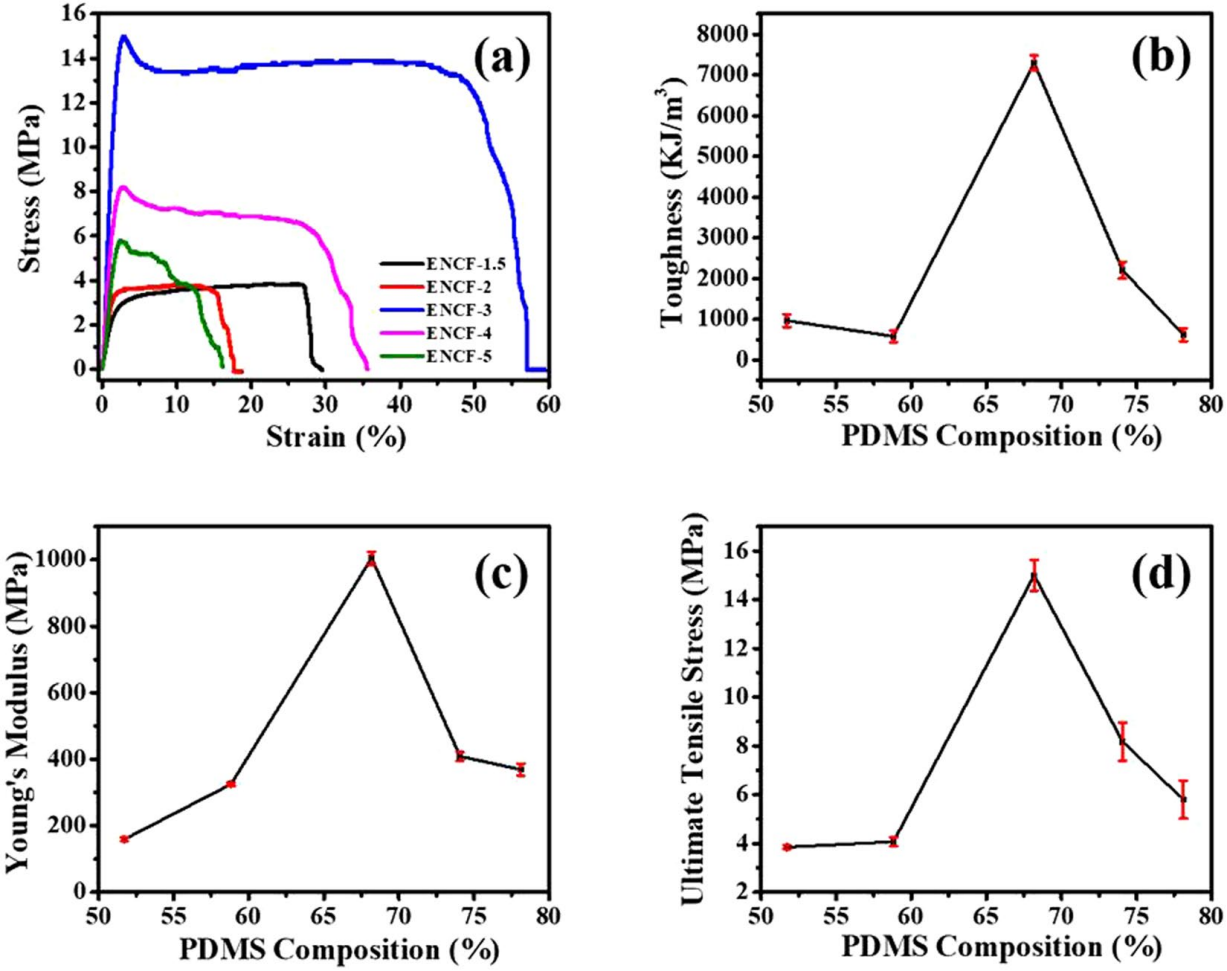
(**a**) Stress-Strain curves of different mats. Dependence of (**b**) toughness (**c**) Young’s moduli, and (**d**) ultimate tensile stress of composite mats on the composition of the PDMS incorporated inside the polymeric mats. There are error bars displayed in (**b**–**d**). However, (**a**) shows the curve from a representative run. The error bars in 6 (**b**–**d**) are obtained from multiple such runs.

One of the motivations behind this work has been to improve the mechanical properties of PVA films those incorporated fatty acid mixtures and exhibited thermoregulatory behavior owing to the eutectic phase behavior exhibited by these mixtures. The electrospun mats of PVA incorporating lauric and stearic acid mixtures in ref.^29^ showed poor mechanical strength, especially at the eutectic composition. In this work, PDMS has been incorporated in the mats containing pure stearic acid to impart mechanical integrity to these mats and to control their hydrophilicity for filtering applications. Performance of these mats in regards with membrane filtering is discussed in the last section of filtration behavior. In this section we present results on the mechanical characterization of these mats performed using tensile testing and dynamical mechanical analysis (DMA). The stress-strain curves along with the trends in the values of Young’s modulus, ultimate tensile stress (UTS), and toughness with increasing PDMS fraction are presented in Fig. 6. A non-monotonic trend in the mechanical properties is evident. Toughness and UTS peaks out in ENCF-3 as shown in Fig. 6(b). Young’s modulus is highest in ENCF-3 (Fig. 6(c)). It is seen that the mechanical behavior typically improves up to the sample ENCF-3 and then starts to deteriorate (Fig. 6(d)). The same trend is also shown by the DMA data as discussed later. In addition, the value UTS obtained here for ENCF-3 mats is nearly twenty-six times larger than the typical values obtained by Gupta *et al*.^29^ The observed trend in the mechanical behavior is consistent with the proposed picture of molecular processes. The crystalline PVA in the fibers provide the most important contribution to the mechanical integrity of the mats. The stearic acid mediated binding of PVA and PDMS keeps on improving the mechanical strength. In low PDMS fraction mats, the stearic acid is present in crystalline forms as suggested by the XRD and DSC data discussed above. These crystalline domains form inclusions in the parent crystalline matrix of PVA. These inclusions give rise to stress concentration and thereby poorer mechanical strength. As PDMS fraction is increased, the stearic acid is better dispersed and as a result the mechanical strength improves. At sufficiently high PDMS fraction, the extra un-bound PDMS chains exist in liquid-like ordering which leads to reduction in strength for PDMS fractions in samples ENCF-3 onwards. The data obtained for various mechanical parameters using UTS is presented in Table 2. These data reconfirm the observation that an optimum PDMS fraction is necessary to obtain mats with the best mechanical property. Fabricating nanofibrous mats of PDMS has been particularly difficult owing to the very low T_g_ and absence of entanglements in PDMS chains. In this regard, our method also offers a route to fabricate PDMS rich nanofibrous mats with respectable mechanical strength^18,23,47^.

**Table 2:** Ultimate Tensile Strength Data.

Sample	Ultimate tensile strength (MPa)	Young’s modulus (MPa)	Toughness (KJ/m^3^)
ENCF-1.5	3.85 ± 0.08	159 ± 5.51	964.55 ± 158.76
ENCF-2	4.07 ± 0.18	325 ± 5.33	579.81 ± 148.67
ENCF-3	15 ± 0.64	1005 ± 19.30	7294.44 ± 186.98
ENCF-4	8.18 ± 0.77	409 ± 12.68	2205.62 ± 208.49
ENCF-5	5.8 ± 0.77	369 ± 18.59	621.33 ± 157.11

### Dynamical Mechanical Analysis (DMA)

Dynamical mechanical analysis is a frequently utilized tool in understanding and optimizing the mechanical and thermal behavior of composites^48,49^. The mechanical characterization by UTS is supplemented here by the dynamic mechanical analysis (DMA) study. In our samples, DMA moduli are nearly double than the Young’s moduli measured by the tensile test and presented in the previous section. We attribute this difference to the fact that UTS measurements are performed in open ambience whereas DMA experiments are performed in a closed chamber with humidity control. Thus UTS measurements are expected to give smaller moduli because of moisture uptake by the samples.

**Figure 7:**
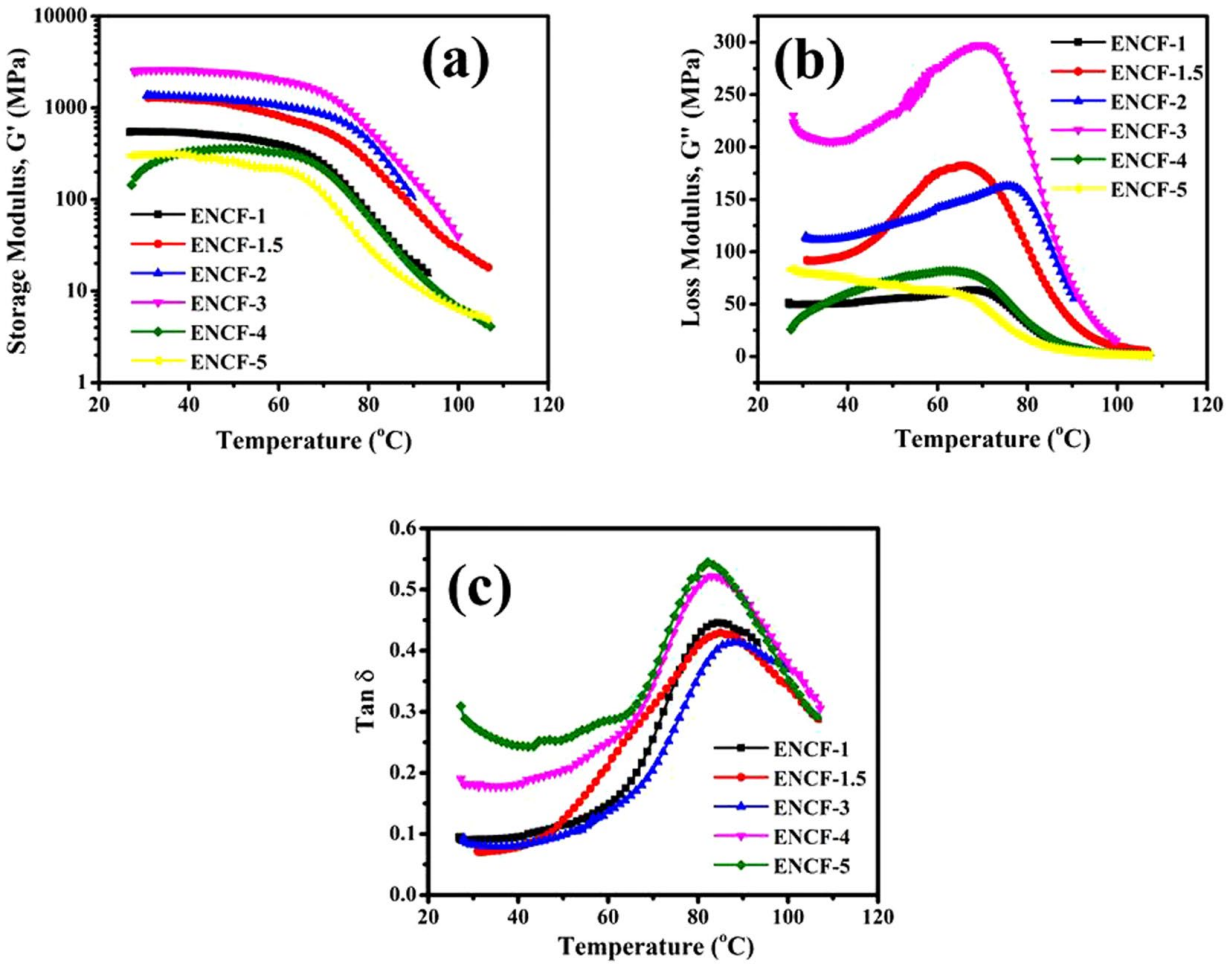
Temperature sweep DMA results for various mats (**a**) storage modulus; G’, (**b**) loss modulus; G” and (**c**) damping factor; tan δ.

**Figure 8:**
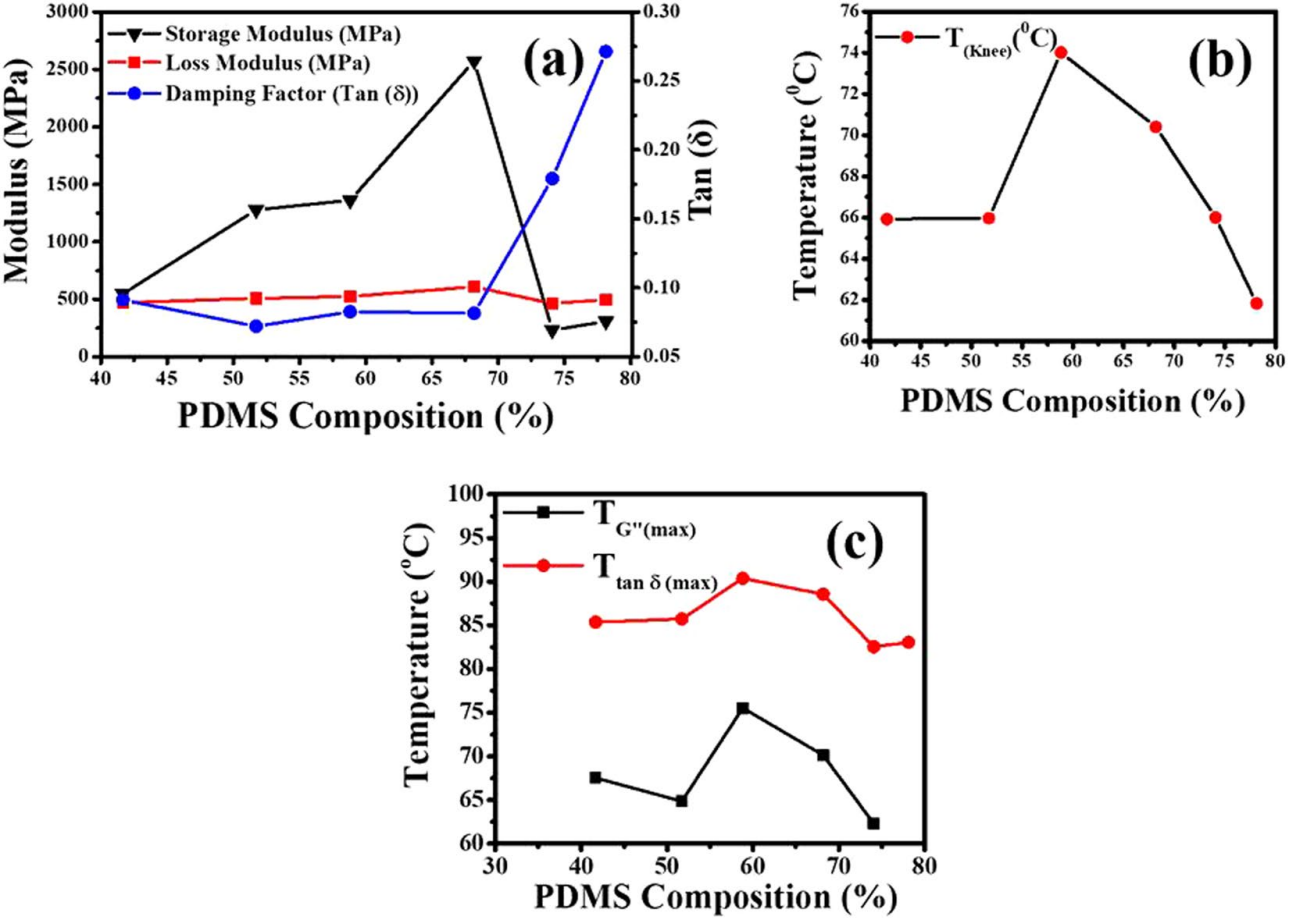
(**a**) Variation of storage and loss moduli, and damping factor at 30 °C, with PDMS fraction in the mats. (**b**) Variation of “knee-temperature”, with PDMS fraction in the mats. (**c**) Dependence of temperatures at which G” and tan δ is maximum, with PDMS fraction in the mats.

DMA measurements use low amplitude oscillations and reveals the viscoelastic nature of the material. We have performed the DMA in stretch mode at 1 Hz with temperature sweep from room temperature to 100 °C. The variations in storage modulus, loss modulus, and the loss tangent (tan δ) are presented in Fig. 7. No perceptible change in mechanical behavior of the as-spun mats is observed at the solid-solid transition temperature recorded in DSC measurements presented earlier. This also confirms that the broad DSC peak in each mat corresponds to regrowth of stearic acid crystallites and a solid-solid transition. A sharp decrease in the values of G’ is observed at temperature near 65 °C which we refer here as the ‘knee’ temperature (*T*_knee_) as shown in Fig. 8(b). Knee temperature is measured in each case by drawing the tangents to the nearly horizontal low temperature segment and the dipping segment of the curves, and finding out their intersection point.

Figure 8(a) presents the G’, G” and tan δ at 30 °C in various samples. We note that in samples with PDMS fraction in excess of 59%, the loss modulus is higher than the storage modulus. This happens because the excess PDMS chains remain in liquid state. A good measure of the tendency of viscous dissipation in a medium is tan δ, whose values at 30 °C shown in Fig. 8(a). A dramatic rise in the value of tan δ is observed in samples with PDMS content above 68%. Higher lossiness at room temperature is attributed to those PDMS chains which are not incorporated with the PVA chains due to short supply of SA. As a result, these ‘unutilized’ PDMS chains are present in liquid like state giving rise to higher damping in the medium. At low PDMS fractions, viscous dissipation is less owing to less number of free PDMS chains and due to SA mediated binding of the PDMS and PVA chains the mat is mostly solid-like.

Figure 8(b) presents the dependence of knee temperature with the PDMS fraction which is noted to be non-monotonic. In smaller PDMS fraction samples, the transition temperature is 66 °C which could be attributed to melting of inclusions of SA micro-crystallite domains. However, beyond an optimal intermediate PDMS fraction of nearly 70%, the stearic acid micro-crystallite domains tend to disappear due to increased participation of the SA molecules in blending the PVA and PDMS chains by getting dissolved in the PDMS as discussed earlier. In these samples the transition in the storage modulus is chiefly due to the glass transition in PVA molecules at 80 °C. At even higher PDMS fractions, the transition temperature is further reduced because of the presence of excess PDMS which helps in bringing down the glass transition temperature in the composite mats.

Behavior of the temperature at which G” becomes maximum is plotted in Fig. 8(c) against the PDMS fraction in the concerned mat and the trend is same as that observed in the knee temperature, *T*_knee_. Variation in the temperature at which loss tangent becomes maximum is shown in Fig. 8(c). This temperature belongs to a transition signaled by the increase in the mobility of long chain polymers. In the present case it corresponds to glass transition in PVA which has been reported to lie between 70–85 °C^48,50,51^. This temperature is around 20 °C above the transition temperature observed in the behavior of the storage modulus (the knee-temperature). PVA chains become more glassier due to presence of SA and PDMS as suggested by the initial rise in the glass transition temperature in Fig. 8(c) obtained from the peak position of the loss tangent. Higher values of temperatures observed for the peak damping-factors as compared to those calculated from transition in the storage modulus (the ‘knee’ temperature) can be explained by noting that storage modulus can be altered due to stearic acid melting transition (near 60 °C) but the glass transition is primarily influenced by the long chain polymer molecules. The effective glass transition eventually starts to decrease due to excess PDMS which plasticizes the composite mat.

### Surface properties and filtration behavior

One of the targeted applications of the PDMS-PVA composite mats synthesized and characterized in this work is filtration of water-in-oil emulsions. In selective membrane applications the nanofibrous structure and the chemical nature of the fiber materials *vis-à-vis* their oleophilicity and hydrophilicity, are two most important design parameters which can be controlled to impart optimal permeability to these mats. Mechanical strength is another important consideration in designing suitable membranes. In an earlier study, vinyl alcohol-ethylene copolymers were considered where variation in the copolymer compositions (in terms of hydrophilic and hydrophobic segments of vinyl alcohol and ethylene) afforded control over the amphoteric nature of the material^52^. In present work, a stearic acid mediated van-der-Waals bonding between PVA and PDMS polymers is exploited to control the hydrophilicity and hydrophobicity of the mats. Here we first show that oleopilicity/hydrophilicity of these mats is monotonically dependent on PDMS fractions. Secondly, we demonstrate that these mats can act as a selective membrane by becoming selectively permeable to oil when exposed to water-in-oil emulsions.

The electrospun mats spun on aluminum foils have a very smooth back surface (the surface adhered to aluminum foil) and a coarse front surface (the surface collecting the fiber while electrospinning). In order to characterize the surface oleophilicity/hydrophilicity we have considered the smooth back surface since the coarseness of the front surface is difficult to control. The RMS heights of the back surface of all the mats were nearly uniform and close to 750 nm as measured by optical surface profilometry (please see supplementary data (Fig. S3). The back surfaces of the mats in contact with the aluminum foil on which the mats are collected, are chosen for characterization of the wetting behavior. Surface roughness can lead to droplets completely wetting the surface or partially wetting it trapping vapor underneath them (Wenzel and Cassie states) giving rise to hysteresis effects in contact angle measurement due to line pinning^53–55^. However, the predominance of hysteresis depends on the statistical nature of the roughness, which can be characterized by the ratio of typical height variation and the associated horizontal correlation length. An analysis of AFM line scan of a representative spot on the mat (Supporting Information, Figs S4 and S5) shows that the ratio of the height variation of the depth (perpendicular to the mat surface) to the in-plane correlation length is very small (on the order of 10^−2^). We have also found that this ratio has values lying in the same range over two widely varying length scales. (Please see Supporting Information, Figs S4 and S5). Therefore, in this work we assume that the effect of the surface roughness on the contact angle can be ignored, and the measured contact angle values arise from the thermodynamics of the wetting phenomena which are intrinsic to the material with a minimal correction due to surface roughness.

**Figure 9:**
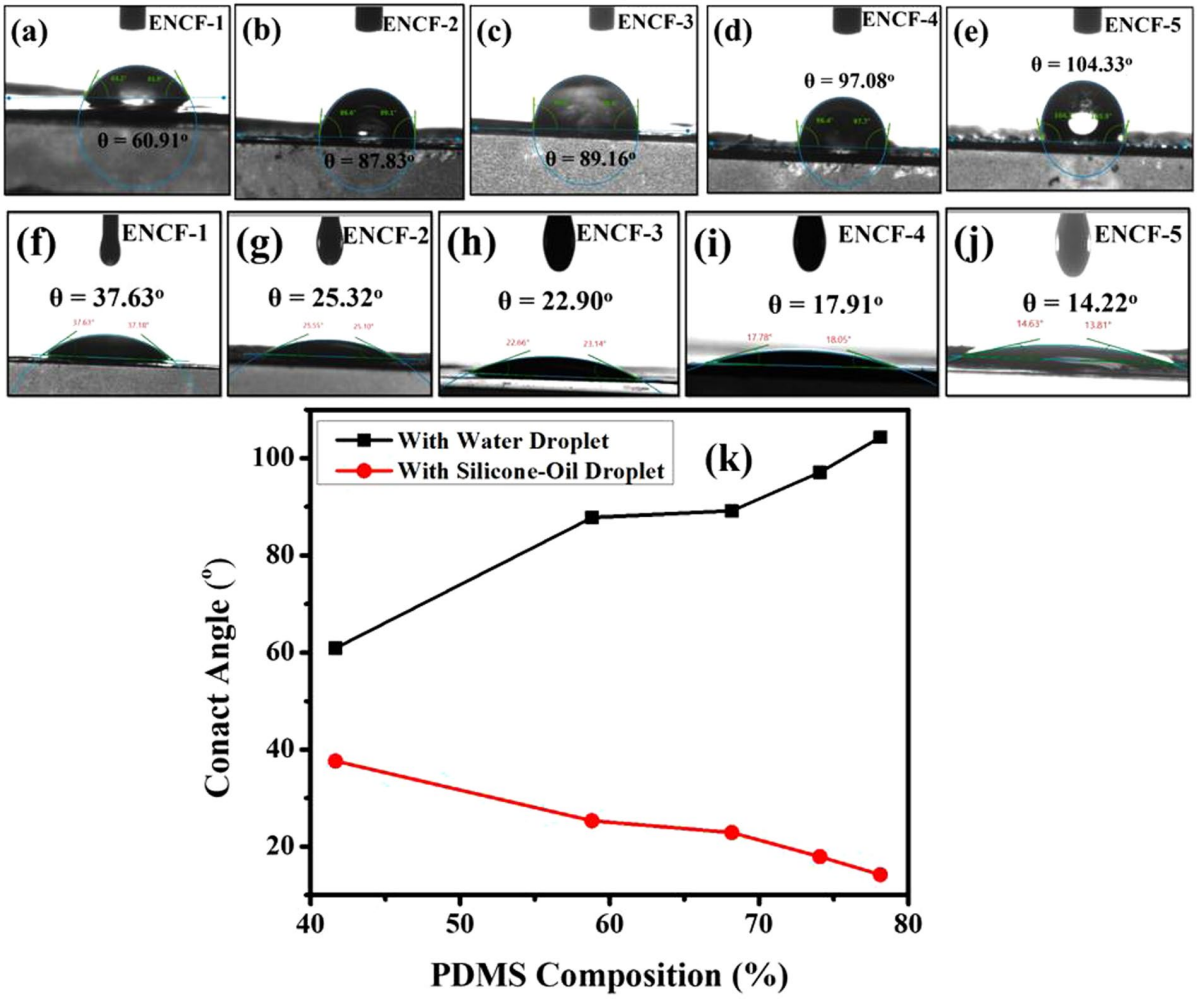
Top panel (a–e) (Water droplet-ENCF interface); Bottom Panel (e–j) (silicone-oil droplet-ENCF interface) and (**k**) comparative data plots of surface wettability towards silicone oil and water at various composition of PDMS.

**Figure 10:**
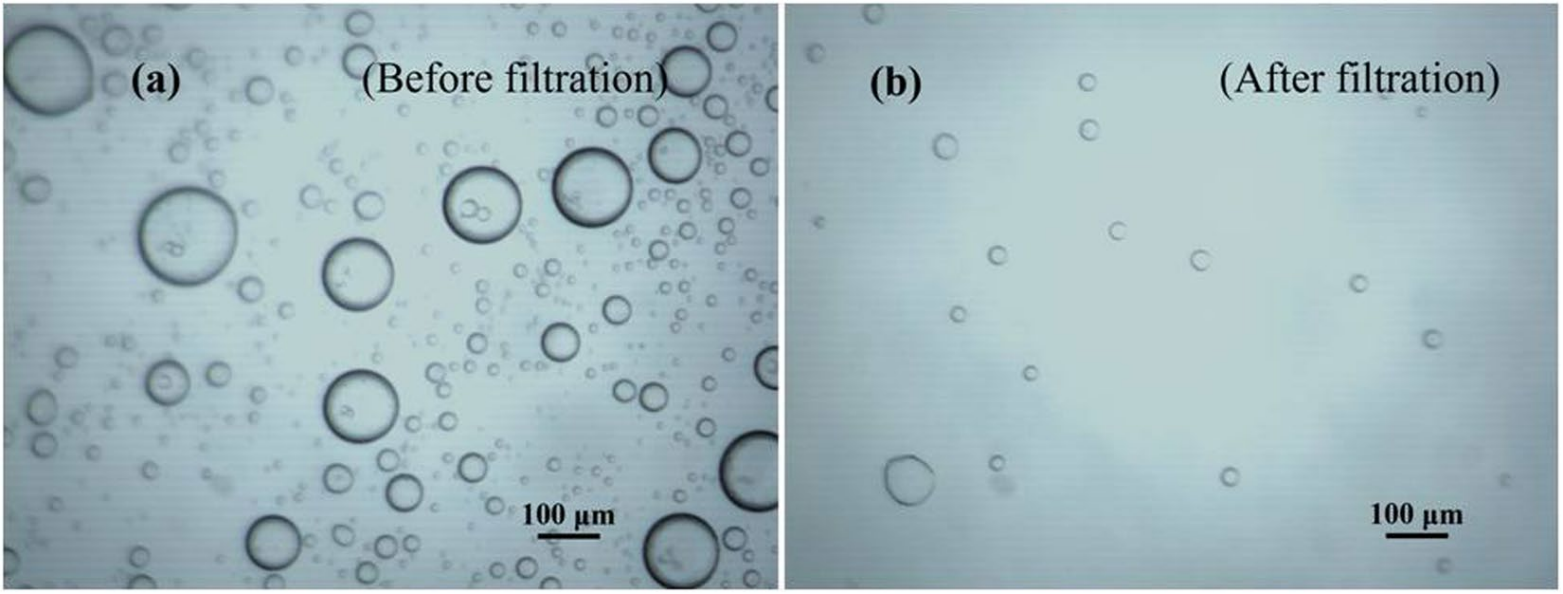
Optical micrograph of oil rich emulsion (water-in-oil) (70%) (**a**) before filtration and (**b**) after filtration by using ENCF-3 membrane.

The contact angles of silicone oil drop and water drops were measured on the back surfaces and the results for different mats with varying PDMS fractions are presented in Fig. 9. It is to be mentioned here that on oleophilic mats, the water droplets stayed for sufficiently long times and allowed contact angle measurements in static conditions. On the other hand, the oil droplets on the same mats disappeared quickly (in less than a second, please see supplementary data, Fig. S1). However, during the fast disappearance, no significant alteration in the contact angle was observed in the snapshots of the droplet. Hence the contact angles reported here with oil droplets pertain to a given snapshot of the droplets while they spread out. As is evident from the contact angle measurements, water-droplet contact angle gradually increases and silicone oil contact angle decreases, that is, the mats keep becoming less hydrophilic and more oleophilic as PDMS content is increased. In addition, it is highly permeable to oil droplets as compared to water droplets as concluded from oil droplets disappearing much faster as opposed to long standing water droplets. This particular property of the mats can be exploited for filtering the oil-water emulsions^56^. We have prepared a water-in-oil emulsion with oil being a majority phase (70%). Before placing a drop of this emulsion, the microscopic image of these emulsions appear as shown in the Fig. 10 wherein the droplets are the water phase and oil is the continuous phase. The microscopic image of the permeate shown in the Fig. 10 clearly establishes that these mats can successfully filter oil selectively from water-in-oil emulsions. That the permeate is oil rich and droplets in Fig. 10(b) are those of water was corroborated by two methods. In the first method, which is by visual inspection, the apparent color of the permeate is found close to that of the oil used originally to make parent emulsions (Fig. S6 in the ‘Supporting Information’). In the second method, the aqueous solution of the rhodamine B (Rh-B) (a dye that easily dissolves in water but doesn’t dissolve in oil), is used to form the parent emulsion with the diesel oil. UV-visible absorption spectra of the following samples were recorded and mutually compared (Fig. S7 in ‘Supporting Information’): (i) pure diesel oil, (ii) aqueous solution of Rh-B, (iii) permeate from the emulsion prepared using aqueous solution of Rh-B, and (iv) permeate from the emulsion prepared without using Rh-B. Since Rh-B associates with water and not with oil, therefore its signal in the UV-Vis spectra is taken to imply the presence of water molecules in the sample. As seen from the spectra (Fig. S7 in ‘Supporting Information’), the strong absorbance of the aqueous Rh-B solution in the visible range is not present either in the pure diesel oil or any of the permeates. Similarity of the absorption spectra of the permeates with the pure oil and absence of Rh-B signal in the permeates (obtained even from those emulsions prepared using aqueous Rh-B solutions) suggest minimal presence of water molecules in the permeates. Hence the permeates are concluded to be rich in oil with water as the minority phase.

We do not have a conclusive model to explain the selective permeability observed in these membranes as yet, but a qualitative explanation can be offered as follows. The mats are formed of PDMS rich material with intermediate pore spaces. The water droplets present in the emulsion are stabilized by the CTAB at the micellar interface and the micelles are dispersed in oil matrix. The PDMS material is oleophilic and water-carrying micelles surrounded with oil molecules is suspended in the inter-fiber pore spaces due to capillary action, and thereby transmission of water across the membrane is blocked. The remaining oil molecules permeate through the PDMS rich fiber material since latter is oleophilic in nature, and diffuse across the membrane. Experimental studies to validate this proposed mechanism are under progress.

## Conclusion

In this work we have shown that stearic acid when mixed with PVA and PDMS, acts like a binding agent between the two polymers and helps in successfully electrospinning a composite nanofibrous mat rich in PDMS content. This simple method carries importance in view of the fact that electrospinning of PDMS has proved extremely difficult due to its low T_g_. The properties of the mats are seen to vary non-monotonically with PDMS fraction: mats with an optimal PDMS fraction range of around 60% shows the best mechanical properties desirable for membrane applications. Based on the data from morphological, structural, thermal, and mechanical characterizations we have outlined a model for the molecular processes which may lead to the observed non-monotonic behavior of the mats. The hydrophilicity and oleophilicity of these mats can be successfully controlled. The PDMS-PVA-SA composite mats show a preferential permeability towards oil when exposed to an oil water emulsion, thus proving to be a suitable candidate membrane material for demulsifying and filtering water-in-oil emulsions.

## Electronic supplementary material

Supporting Information
