# Supplementary material for: PVA-PDMS-Stearic acid composite nanofibrous mats with improved mechanical behavior for selective filtering applications
Source: Sci Rep. 2018 Oct 30;8:16038. doi: 10.1038/s41598-018-34440-5 (PMC6207706; doi:10.1038/s41598-018-34440-5)
Supplement: Supplementary file 1 — Supporting Information [file 41598_2018_34440_MOESM1_ESM.doc]

“Supporting Information” for

**PVA-PDMS-Stearic acid composite nanofibrous mats with improved mechanical behavior for selective filtering applications**

**Shama Perween^a^, Ziyauddin Khan^b,c^, Somendra Singh^a^, Amit Ranjan^a^***

*^a^Rajiv Gandhi Institute of Petroleum Technology, Jais, Uttar Pradesh, India.*

*^b^School of Energy and Chemical Engineering, Ulsan National Institute of Science and Technology (UNIST), Ulsan, Republic of Korea.*

*^c^Present address: Laboratory of Organic Electronics, Department of Science and Technology, Linköping University, SE-601 74 Norrköping, Sweden.*

**CORRESPONDING AUTHOR: Amit Ranjan* (*Email:*** [***aranjan@rgipt.ac.in***](mailto:aranjan@rgipt.ac.in) ***)****

**
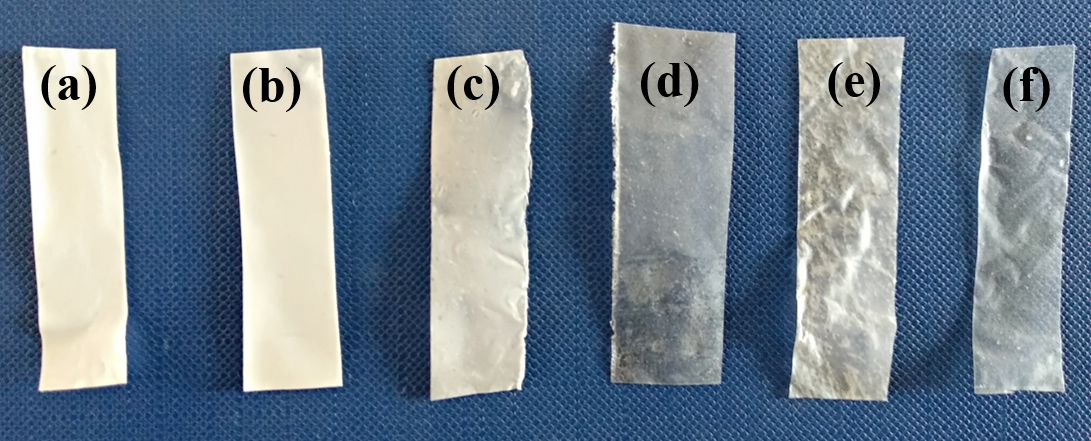
**

**Figure S1. Photographs of ENCF mat strips with variable composition of PDMS (top-view) (a)ENCF-1 (b) ENCF-1.5 (c) ENCF-2 (d) ENCF-3 (e) ENCF-4 and (f) ENCF-5.**


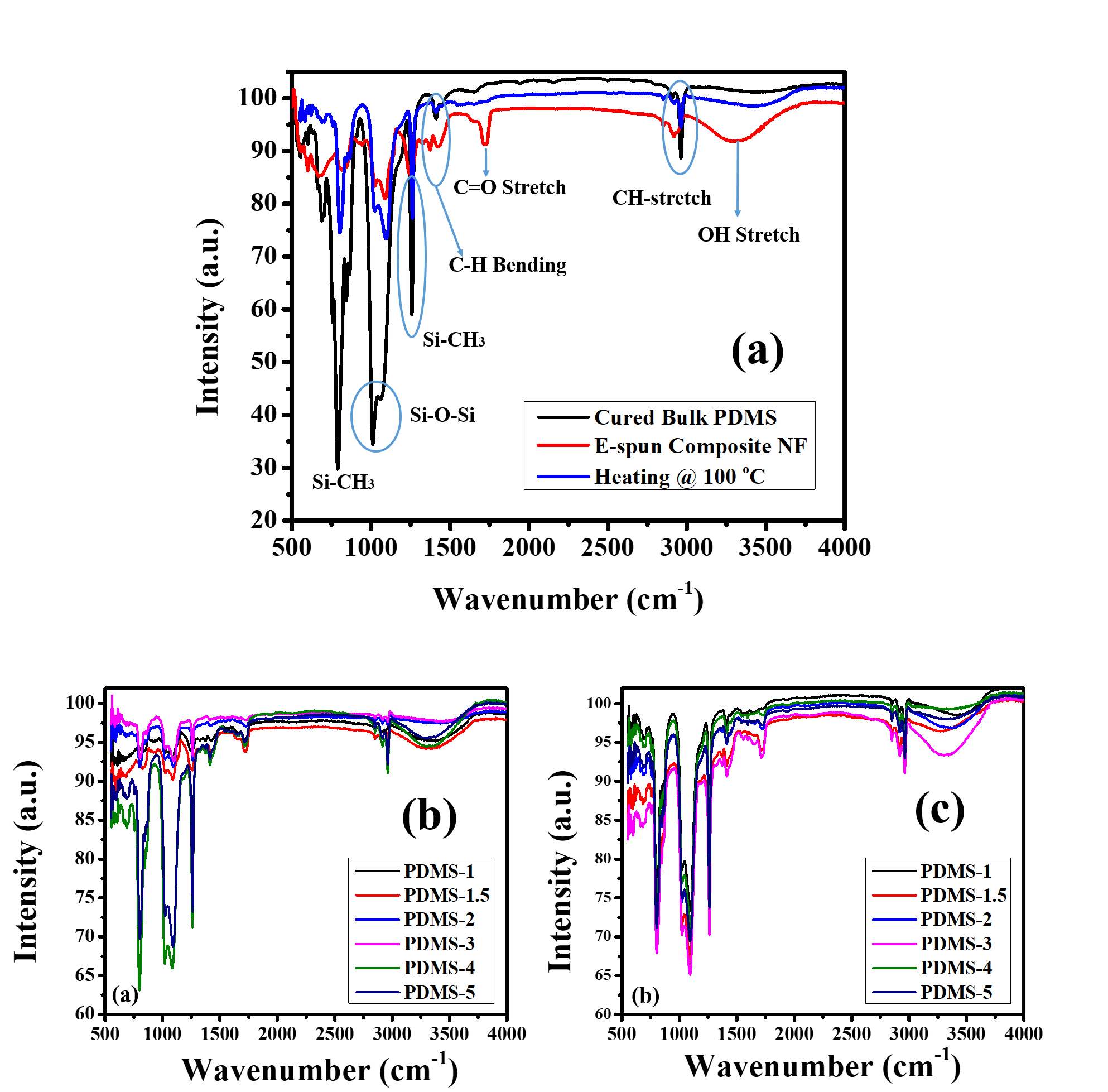


**Figure S2.** (a) FT-IR spectra of the as spun ENCF mats compared with cured bulk PDMS (b) before and (c) after thermal cycling at 100 ^o^C.

**
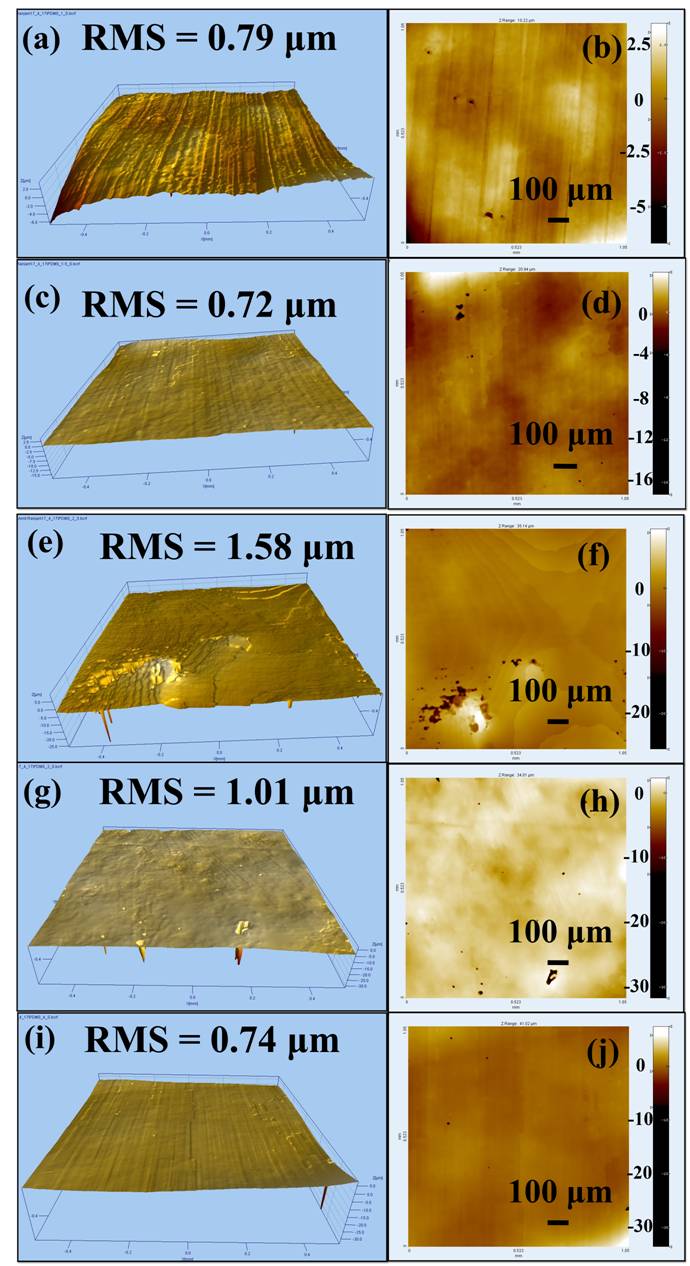
**

**Figure S3.** Optical surface profilometer image of ENCF mats with increasing PDMS composition (%). Left side panel from top to bottom (a, c, e, g, i) shows 3-D and right side panel (b, d, f, h, j) 2-D images.


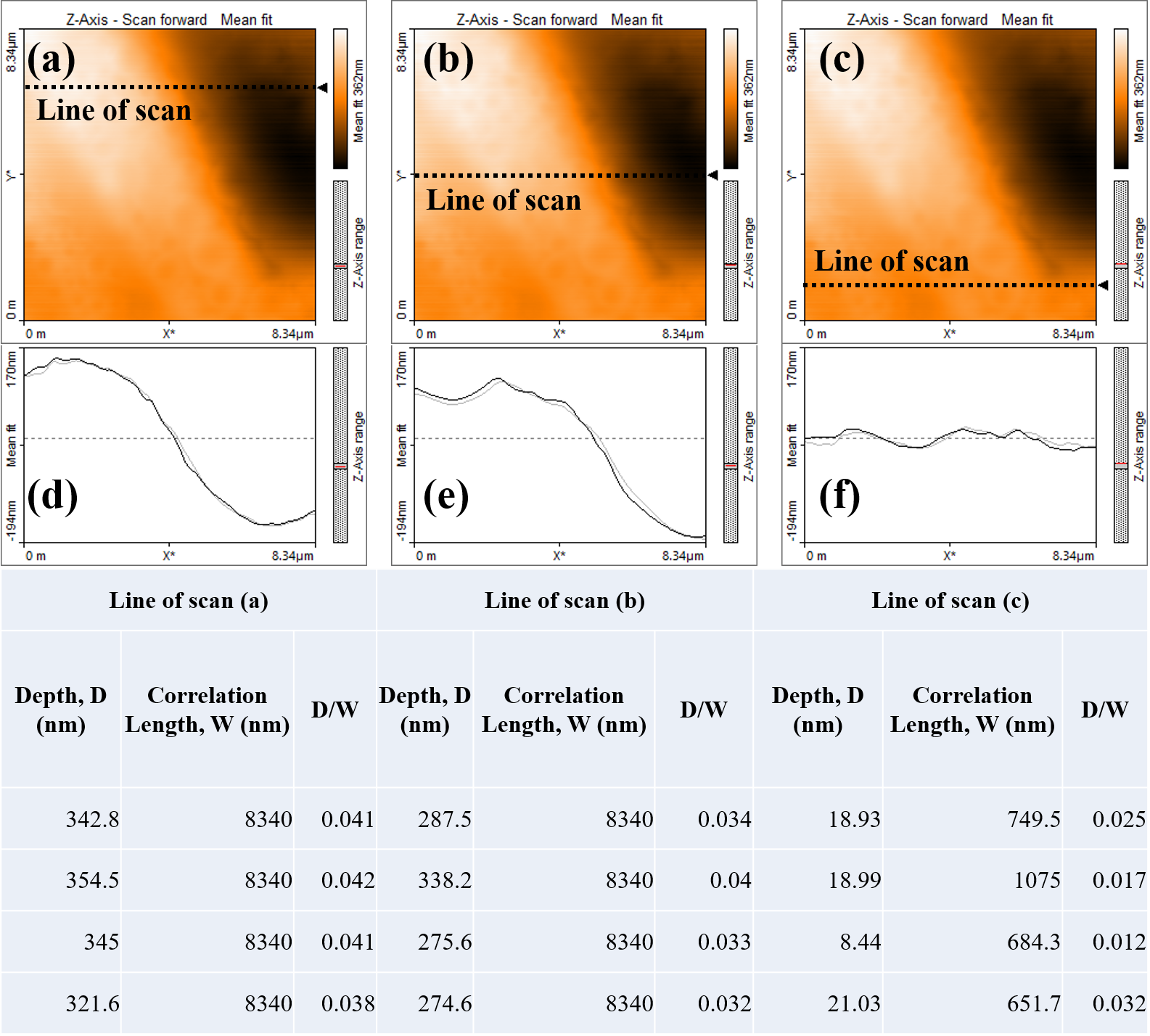


**Figure S4.** (a), (b) and (c) presents the AFM images with three different lines of scan. (d), (e) and (f) represent the height variation along the corresponding scan-lines. The table lists the values of height variation, the corresponding correlation length and their ratios, in respective columns. These values are obtained from visual inspection. The length of the line scan is 8.34 μm.


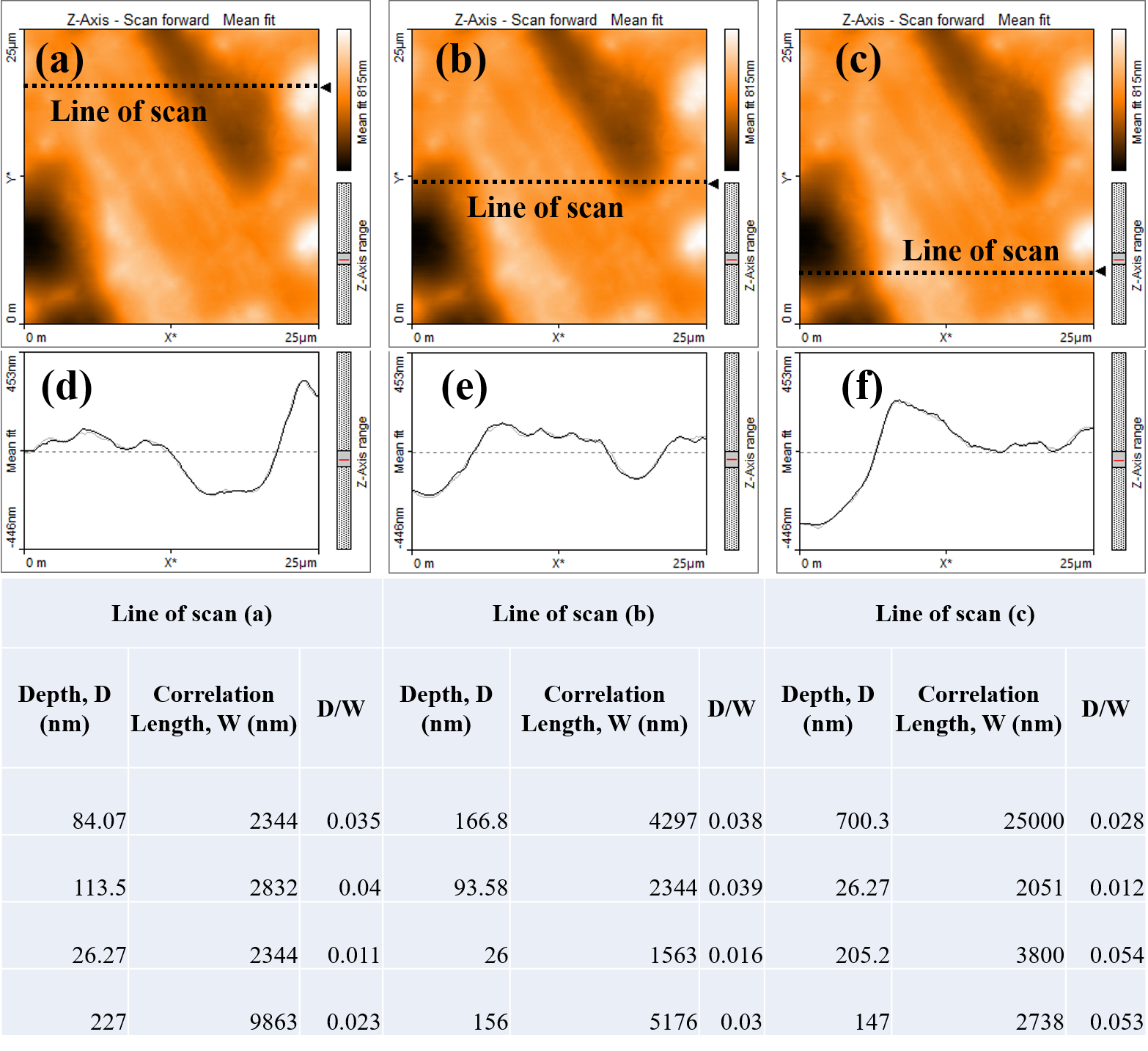


**Figure S5.** (a), (b) and (c) presents the AFM images with three different lines of scan. (d), (e) and (f) represent the height variation along the corresponding scan-lines. The table lists the values of height variation, the corresponding correlation length and their ratios in respective columns. These values are obtained from visual inspection. The length of the line scan is 25 μm.


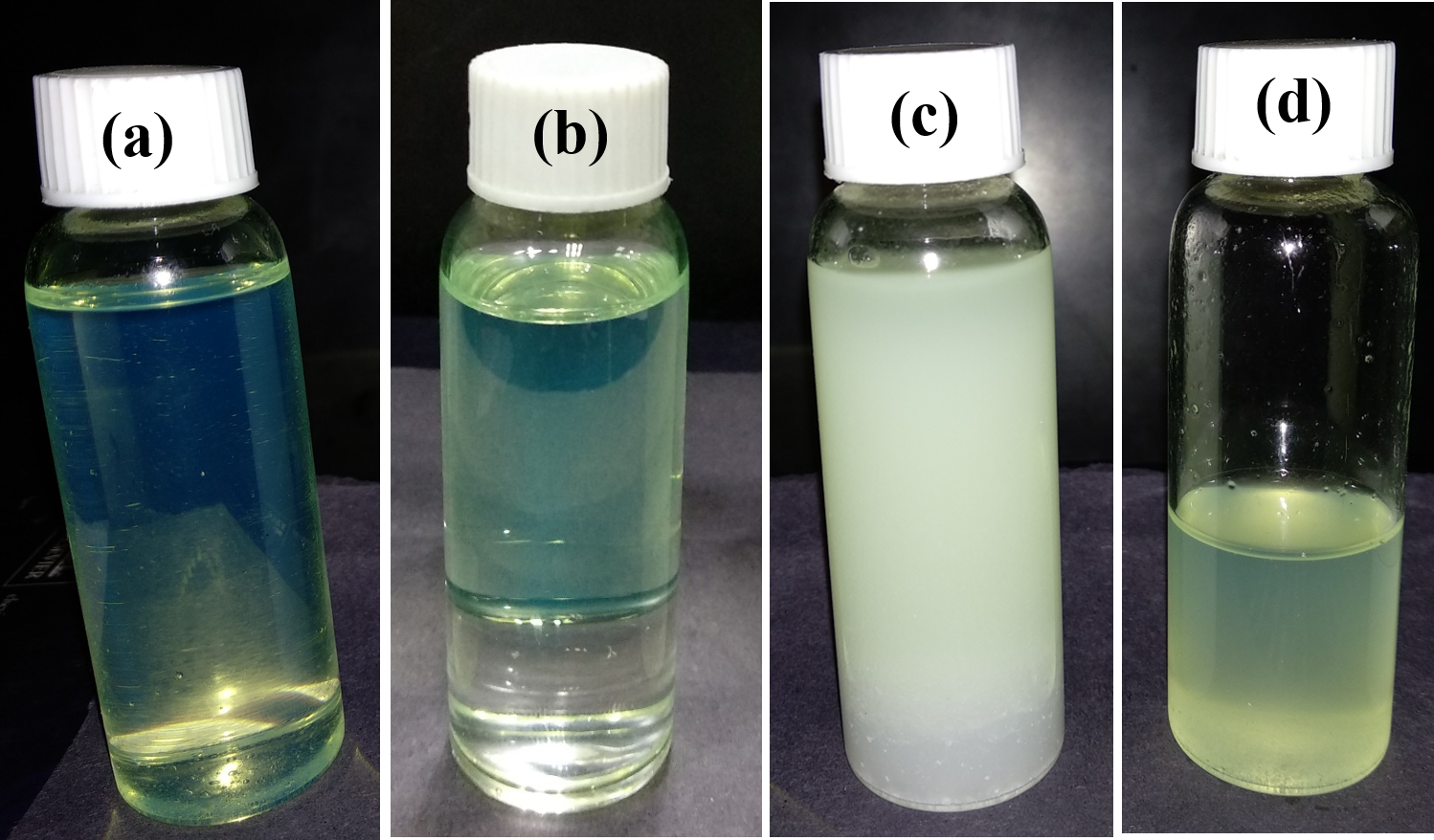


**Figure** **S6.** The digital images of (a) pure diesel oil, (b) phase-separated water and oil with a ratio of 3:7, (c) resulting water-in-oil emulsion before filtration, prepared using CTAB surfactant, and (d) permeate from the emulsion after filtration.


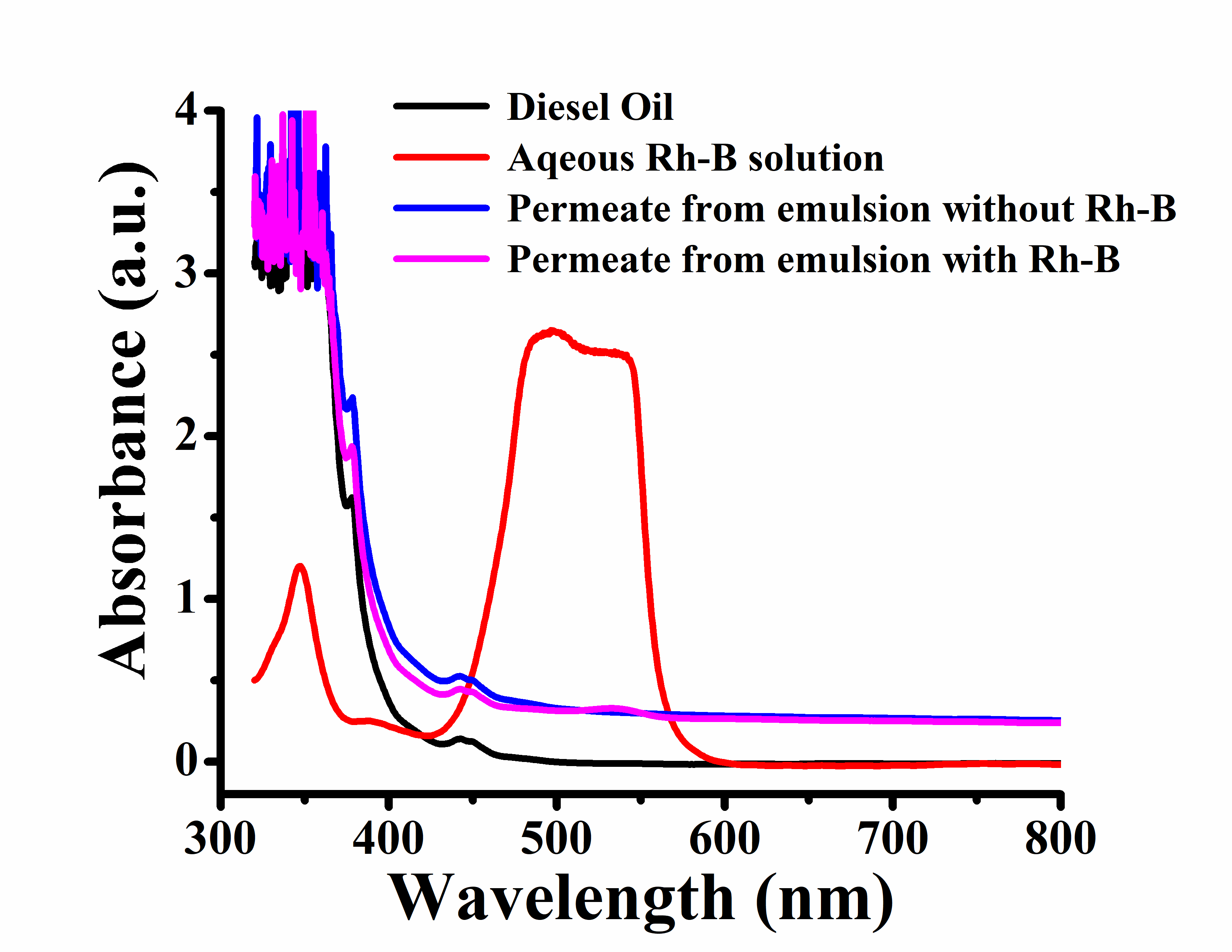


**Figure S7.** A mutual comparison of the UV-Visible absorbance spectra of pure diesel oil, aqueous solution of Rh-B dye, and permeates obtained from the emulsions *with* and *without* Rh-B dye.
